# Acceptability of community health worker and peer supported interventions for ethnic minorities with type 2 diabetes: a qualitative systematic review

**DOI:** 10.3389/fcdhc.2024.1306199

**Published:** 2024-05-21

**Authors:** Vivene Grant, Ian Litchfield

**Affiliations:** ^1^ Birmingham Medical School, College of Medical and Dental Sciences, University of Birmingham, Birmingham, United Kingdom; ^2^ Institute of Applied Health Research, College of Medical and Dental Sciences, University of Birmingham, Birmingham, United Kingdom

**Keywords:** type 2 diabetes, self-management, community health, health inequalities, ethnic minorities

## Abstract

**Objective:**

Ethnic minority groups in high income countries in North America, Europe, and elsewhere are disproportionately affected by T2DM with a higher risk of mortality and morbidity. The use of community health workers and peer supporters offer a way of ensuring the benefits of self-management support observed in the general population are shared by those in minoritized communities.

**Materials and methods:**

The major databases were searched for existing qualitative evidence of participants’ experiences and perspectives of self-management support for type 2 diabetes delivered by community health workers and peer supporters (CHWPs) in ethnically minoritized populations. The data were analysed using Sekhon’s Theoretical Framework of Acceptability.

**Results:**

The results are described within five domains of the framework of acceptability collapsed from seven for reasons of clarity and concision: *Affective attitude* described participants’ satisfaction with CHWPs delivering the intervention including the open, trusting relationships that developed in contrast to those with clinical providers. In considering *Burden* and *Opportunity Costs*, participants reflected on the impact of health, transport, and the responsibilities of work and childcare on their attendance, alongside a lack of resources necessary to maintain healthy diets and active lifestyles. In relation to *Cultural Sensitivity* participants appreciated the greater understanding of the specific cultural needs and challenges exhibited by CHWPs. The evidence related to *Intervention Coherence* indicated that participants responded positively to the practical and applied content, the range of teaching materials, and interactive practical sessions. Finally, in examining the impact of *Effectiveness and Self-efficacy* participants described how they changed a range of health-related behaviours, had more confidence in dealing with their condition and interacting with senior clinicians and benefitted from the social support of fellow participants and CHWPs.

**Conclusion:**

Many of the same barriers around attendance and engagement with usual self-management support interventions delivered to general populations were observed, including lack of time and resource. However, the insight of CHWPs, their culturally-sensitive and specific strategies for self-management and their development of trusting relationships presented considerable advantages.

## 1 Background

**Figure 1:**
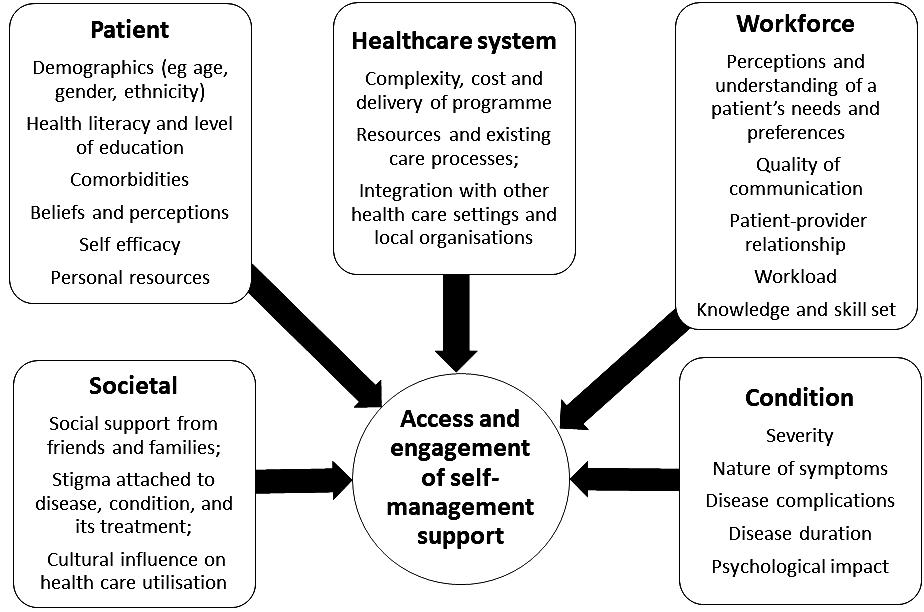
Factors affecting access and engagement with (diabetes) self-management programmes (15).

Type 2 diabetes mellitus (T2DM) is a prominent global health challenge impacting 536.6 million adults worldwide (1). Ethnic minority groups in high income countries in North America, Western Europe, Australia and New Zealand are disproportionately affected by T2DM and also have a higher risk of mortality and morbidity (2–7). Supported self-management that helps control blood sugar levels and sustain positive health and lifestyle behaviours is an internationally recognised method of improving the prognosis for those with T2DM (8, 9). However, in developed countries, the benefits of self-management are not realised in minoritized communities more vulnerable to the cultural and social barriers which impact engagement with diabetes care more broadly, and specifically with self-management support (10, 11). These barriers include health literacy, cultural stigma, a lack of personal resource, the severity of the condition, and broader factors involving the healthcare system and the interaction with care providers (10, 12–15) (as summarised in **Figure 1**).

With the increasing prevalence of T2DM in minoritized communities, it is important to identify and understand which components of self-management interventions might be best placed to address these barriers (15, 16). One approach that has shown promise in T2DM, and other long-term conditions, is the use of individuals linguistically, experientially and ethnically indigenous to target communities in the delivery of diabetes self-management advice and education (DSME) and other aspects of self-management support (4, 17–22). These individuals have been employed in a range of roles with various names including community health workers, lay health workers, peer educators, and peer mentors, and receive varying levels of training (17–19, 23, 24). For the purposes of this review, we use the collective term community health worker and peer supporter or educator (CHWP) defined as: “non-professional workers who are from or understand local communities, and deliver structured community-based support or education for diabetes self-management having received intervention-specific training” (25).

Despite inconsistent empirical evidence of CHWPs’ direct effect on diabetes outcomes, early indications are that CHWPs offer a promising means of improving HbA1c control (21, 26) as well as psycho-social benefits of increased self-efficacy and acceptance (27). However, if this impact is to be optimised in minoritized populations, it is important to understand participant views on CHWPs and use these to inform future iterations (28–32).

This qualitative systematic review collates and analyses qualitative evidence from participants in diabetes self-management interventions for ethnic minorities delivered by CHWPs. To structure our findings and analysis we used Sekhon’s Theoretical Framework of Acceptability (TFA) which was developed to explore acceptability of health care interventions from the perspectives of providers and patients (33). We described participant experiences and perspectives of care interventions within its domains including the burden of participation, intervention coherence, and perceived effectiveness (33). This has allowed us to identify several key factors influencing the success of CHWP-led interventions in minoritized communities and enabled the creation of a series of recommendations for future implementation.

## 2 Methods

### 2.1 Study design

A systematic review of qualitative data describing participant experiences of CHWP-led T2DM self-management interventions in minoritized populations. Eligibility criteria and search terms were developed using the SPIDER (Sample, Phenomenon of Interest, Data, Evaluation, Research Type) tool which is tailored specifically to the screening of qualitative research (34) (see **Supplementary File 1**). The findings are presented within an adapted version of Sekhon’s Theoretical Framework of Acceptability (33).

### 2.2 Theoretical framework of acceptability

**Figure 2:**
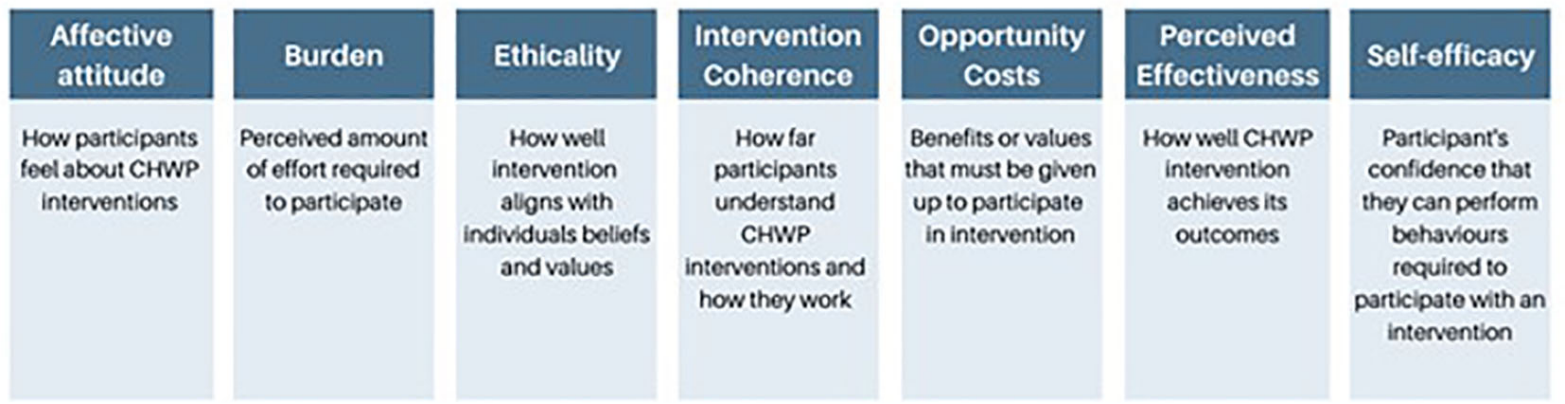
Theoretical Framework of Acceptability (TFA) and Definitions (33).

The TFA was developed by Sekhon et al. firstly by producing an overview of reviews that defined, theorised or measured the acceptability of healthcare interventions (33). This was then used to develop the theoretical framework by defining acceptability, describing its properties and scope, and then identifying component constructs and empirical indicators (33). The final TFA consisted of seven domains: affective attitude, burden, ethicality, intervention coherence, opportunity costs, perceived effectiveness, and self-efficacy (see **Figure 2**). Together they describe whether those receiving (or delivering) an intervention consider it to be appropriate based on anticipated or experiential cognitive and emotional responses (33). It has been successfully used in a range of settings and circumstances including the treatment of HIV (35), breast cancer (36) and mental health (37). The seven domains and their constructs were used to guide the presentation and analysis of our qualitative findings to understand the acceptability of CHWP interventions in ethnic minority groups.

### 2.3 Search strategy

The following five electronic databases were searched in August 2023: MEDLINE, Excerpta Medica dataBASE (EMBASE via Ovid), Cumulative Index to Nursing and Allied Health Literature (CINAHL via EBSCO), PsycInfo and Web of Science. Google Scholar and ProQuest Dissertations & Theses Global were also searched to identify grey literature. The search strategy (See **Supplementary File 2**), including all index terms and identified keywords, was adapted for each specified database. Reference lists of eligible papers were hand searched for additional citations. No date limits were applied as CHWP involvement in diabetes management first appeared in the literature in the 1980s (38). The geographical range of the included studies was restricted to Canada, New Zealand, Australia, Western Europe and the USA as they constitute developed health economies (all be it with different models of healthcare provision) and are resident to sizeable ethnic minority communities i.e. all ethnic groups whose members typically share a combination of characteristics of culture, religion or language, except the white group of western European origin (39, 40).

### 2.4 Study selection

Search results were exported and indexed in EndNote 20 (41). After removing duplicates, studies were screened using Covidence software, a web-based system for managing systematic reviews (42). Studies which met the following criteria were included: (1) Conducted in a high-income western country [as defined by the world bank (43)]; (2) Explored participants’ perspectives using qualitative methods; (3) Interventions (including programmes or elements of programmes) led by CHWPs related to self-management or prevention of T2DM; (4) Ethnic minority adults represented the majority of the cohort explored; and (6) Full-text versions were available in English. Studies were excluded: if (1) the role of the CHWP could not be clearly identified or (2) were aimed at those with type 1 diabetes mellitus (T1DM) or gestational diabetes.

Two independent reviewers including the 1^st^ author carried out title and abstract screening using the above eligibility criteria. Both reviewers received training in qualitative research methods and systematic review screening as part of their intercalated training. Any disagreements were firstly discussed between the reviewers (i.e. VG and FJ) and escalated to IL for a final decision if they could not be resolved. The two reviewers screened full-text versions of studies and excluded papers which did not fulfil inclusion criteria: IL had oversight of the selection process and all papers included in the review were consensually agreed by both authors.

### 2.5 Data extraction and quality appraisal

Data were first extracted on general study characteristics and key intervention components. Study data were imported into NVivo 1.0 software for analysis (44). Where studies employed mixed methods, only qualitative data were extracted. The data included in the analysis consisted of *verbatim* quotes from participants of individual studies and authors’ interpretations of findings.

The Critical Appraisal Skills Programme (CASP) Qualitative Checklist was used by two independent reviewers to appraise the limitations and strengths of each study’s methodological reporting (**Supplementary File 3**) (45). No articles were excluded based on reporting quality as all studies enhanced the conceptual richness of the final synthesis. In mixed method studies, only qualitative aspects were appraised.

### 2.6 Data synthesis

A framework-based approach was used combining both deductive and inductive analyses to populate the Theoretical Framework of Acceptability (TFA) (33, 46). Analysis consisted of two main stages: First *Deductive Coding* was applied which involved familiarisation with the data set and allocating the data into the most appropriate TFA domains. Second, we used an iterative process of *Inductive Coding* whereby similar codes within each domain were grouped leading to the final set of domains and constructs.

## 3 Results

### 3.1 Study characteristics

**Figure 3:**
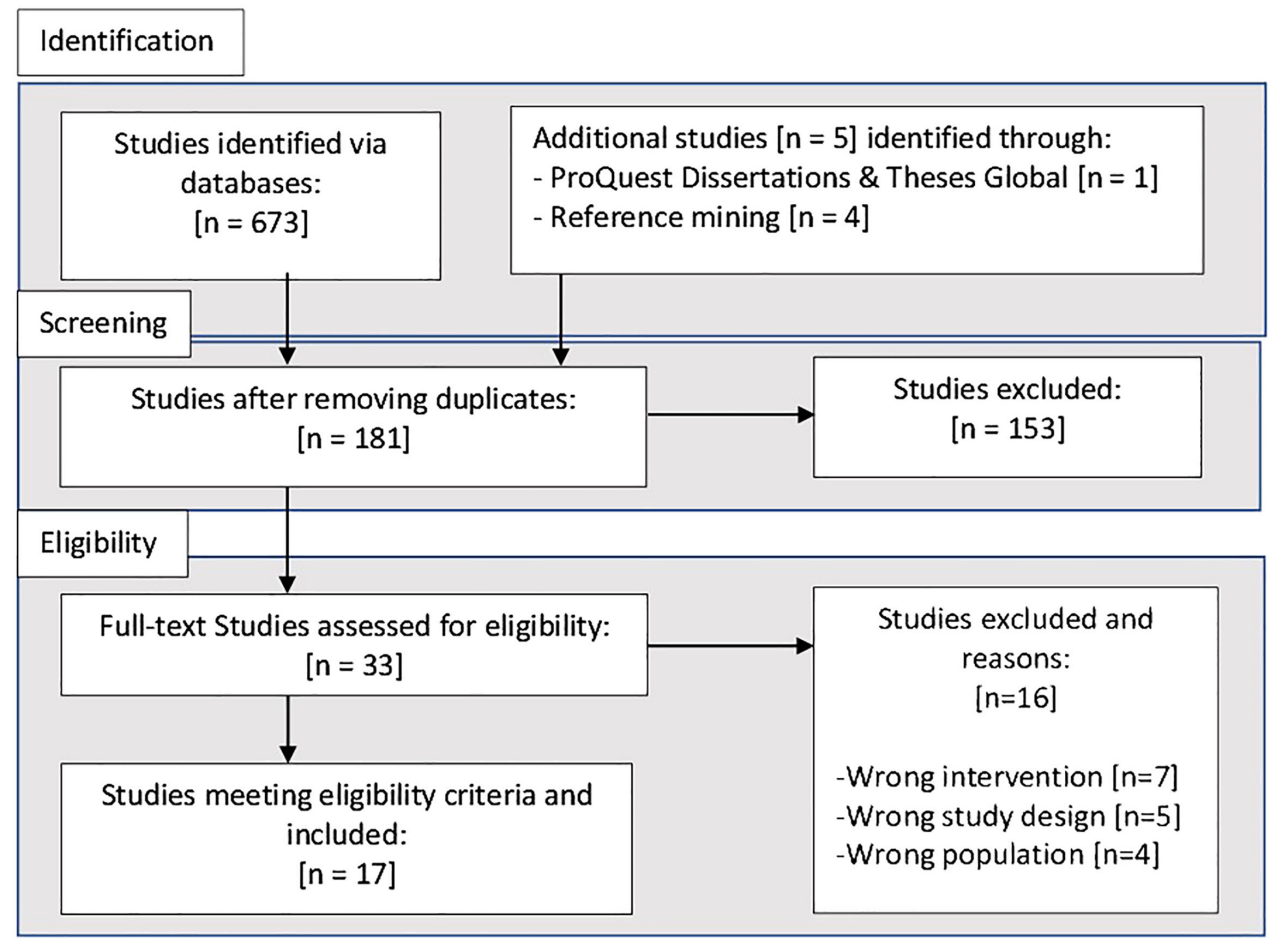
PRISMA diagram.

A total of 17 studies were included in the systematic review. The PRSIMA diagram describing study selection is shown in **Figure 3** (47). In total the views and perceptions of 387 participants were described, with 15 studies conducted in the USA, one in Canada and another in Australia; of these seven focussed on Latino populations (48–54); five African American (55–59); one mixed of both populations (60), one Native American (61), one Punjabi (62); one Aboriginal (63); and one on Pacific Islanders (64). Papers were published between 2009 and 2022. All studies focused on participant perspectives post-intervention and thus assessed retrospective acceptability. Key study characteristics and specific intervention components are summarised in **Table 1**.

**Table 1:** Study characteristics.

Authors *(Date)*	Setting Country *(Venue of intervention)*	Research Aim	Sample Characteristics	Programme title *CHWP Role*	Data collection; analysis	Findings
Castillo et al., 2010a (48)	USA, *(Community Venues* ^a^ *)*	To assess the feasibility of a culturally and linguistic appropriate diabetes education programme led by CHWPs	Latinos with T2DM: (n=15) Female 13, Male 2 Age: 25-85	**Diabetes Empowerment Education Program**; *-Led 10-week bilingual group DSME sessions* *-Encouraged family and friend participation*	2 Focus Groups; Thematic Analysis Exploring increased knowledge, self-efficacy, gender roles, mental health, and bond with CHWP	Significant improvements in HbA1C (P = .001) and in diabetes knowledge, physical activity, healthy eating, and glucose self-monitoring.
Chang et al., 2021 (49)	USA, *(Clinics & Participants’ homes)*	To examine patients’ perceptions of the feasibility, acceptability, and impact of a safety net-based CHWP programme	Adults with T2DM: (n=13). 9 Female, 4 Male Average age: 56 Race/ethnicity: 9 Hispanic/Latino, 4 unreported	**CHW Pilot Intervention**; *-Delivered one-on-one personalised support (help navigate appointments, provide transport)*	Semi-structured Interviews; Grounded Theory Exploring health care access, social needs, and self-efficacy	Participants were satisfied with the intervention, including the usefulness of topics covered, CHWP’s professionalism, knowledge, and engagement.
Deitrick et al., 2010 (50)	USA, *(Clinics)*	To examine the role of a promotora who provides diabetes education to the Puerto Rican community	Latino patients with T2DM: (n=35). 21 Female, 14 Male Age: 40-82	**Learning** **About Diabetes**; *-Led DSME group sessions in Spanish* *-Provided review sessions for illiterate participants*	6 Focus Groups; Grounded Theory Exploring relationship with HCPs, language, and increased knowledge	Participants reported satisfaction with the intervention, increased ability to self-manage diabetes, and strengthened connections with other Latinos with diabetes.
Haltiwanger and Brutus, 2012 (54)	USA, *(Community Diabetes Education Centre)*	To evaluate whether the Peer Support Group can change adherence behaviours in patients with T2DM	Mexican-Americans with T2DM: (n=32). Female NR^a^ Male NR Age: NR	**Bridges Diabetes** **Peer Support Group**; *- Used diabetes workbook on health care beliefs and personal goals to lead group discussion* -*Used stories to empower individuals*	30 Focus Groups; Thematic Analysis Exploring social cost of T2DM, Distrust of HCPs, and patient empowerment	Participants reported a lack of trust in clinicians, increased pressure in social situations, and valued support of peers.
Heisler et al., 2009 (60)	USA, *(Community Venues)*	To explore how a CHWP diabetes programme influences participants’ diabetes management and interactions with HCPs	African Americans and Latinos with T2DM (n=40). 32 Female, 8 Male Age: 38-72 Race/ethnicity: 20 African American, 20 Latino	**Journey to Health**; *- Led DSME* ^a^ *group teaching sessions*	Semi-structured Interviews; Thematic Analysis Exploring information on participants’ relationship with HCPs	Participants felt information was clear. CHWPs were non-judgemental and social support improved self-efficacy including confidence when speaking with clinicians.
Jia et al., 2019 (55)	USA, (Public Library & Telephone)	To determine the intervention feasibility, acceptability and efficacy using quantitative and qualitative methods.	Adults with poorly controlled T2DM: (n=15) Average Age: 56 Race/ethnicity: African American	**CHW Photovoice Intervention**; *-Delivered photovoice sessions followed by group discussion over 3 months* *-Telephone contact between sessions*	Focus groups; Thematic analysis Exploring increased motivation, value of interpersonal relationships, and adherence challenges	No improvement on HbA1c due to lack of dietary knowledge, and frustration towards diabetes self-management.
Joachim et al., 2020 (51)	USA, *(Community Venues)*	To examine readiness of low-income Latinas to adhere to and to adopt preventive behaviours	Latina women at-risk of T2DM (n=40). Age:37-59	**Vida Vibrante Diabetes Prevention**; *- Delivered DSME group sessions* *- Used culturally appropriate teaching aids (‘loteria’ cards)* *- Relayed challenging questions to a physician*	9 Focus Groups and 3 Semi-structured Interviews; Thematic Analysis Exploring social support, family health concerns, and awareness of diabetes complications	Barriers to engagement included built environment, financial constraints, and threat of social alienation. Mitigating factors included physician involvement, and social support.
Lalla et al., 2020 (61)	USA, *(Participants’ Homes)*	To understand the perspective of patients through reported outcomes	Navajo adults with history of T2DM: (n=7). Female 1, Male 6 Average age: 60.1	**Community Outreach and Patient Empowerment**; *-Led one-on-one DSME sessions* *-Used incentives (measuring cups, portion plates, exercise bands)*	Semi-structured Interviews; Thematic Analysis Exploring self-efficacy, health literacy, benefits of family participation, CHWP-patient bond, and life balance	CHWPs were successful in supporting lifestyle changes, helping understand key health indicators, and setting achievable goals. CHWPs made regular visits to educate family members.
McGowan, 2013 (62)	Canada, *(Community Venues)*	To understand participant’s perceptions of the programme benefits	Punjabi adults at high risk of T2DM: (n=34) Female NR, Male NR Age: NR	**Punjabi Chronic Disease Self-Management Program**; *-Delivered bilingual group DSME workshops over 6 weeks*	3 Focus groups Comments on improved HCP communication, improved health, mood, and confidence	There were changes in lifestyle behaviours, improvements in psychological status, self-efficacy, and community involvement.
Okoro et al., 2018 (56)	USA, *(Participants’ Homes & Telephone)*	To identify the important features of a culturally appropriate peer support T2DM programme for African Americans	African Americans with T2DM: (n=20) Female 13, Male 7 Age: 30-82	**Peer Support Programme**; *- Delivered group DSME sessions*	Semi-structured Interviews; Thematic Analysis Exploring factors influencing healthy behaviours, and emotional support	Healthy behaviours were adopted thanks to frequent telephone contact and greater emotional support.
Otero-Sabogal et al., 2010 (53)	USA, *(Clinics & Telephone)*	To improve diabetes self-management by incorporating CHWPs as members of a hospital clinical team	Patients at a Health Care Centre with T2DM (n=31). Female NR Male NR Age: NR Race/ethnicity: Latino population	**HealthFirst**; *-Led individual and group DSME sessions* *-Provided telephone follow-up* *-Worked alongside social worker and doctor*	Satisfaction Survey; Thematic Analysis Exploring relationship between patients and CHWPs	HbA1c control and lifestyle behaviours improved. Patients were satisfied with CHWP support.
Pullen Smith, 2014 (57)	USA, *(Community Venues)*	To understand the challenges of programme implementation from perspectives of Community Health Ambassadors and clients	Residents of a public housing development with T2DM: (n=5) Female 5 Age: NR Race/ethnicity: 4 African American, 1 White	**Community Health Ambassador Program**; *-3 months minimum, delivered group and one-on-one outreach activities (healthy eating, cooking demonstrations)*	Focus group; Thematic Analysis Exploring trust in CHWPs, achieving goals, identifying with group, and inspiration to change	Data suggested improvements in key clinical outcomes and health behaviours. Patients felt that CHWPs were beneficial to individuals and their community.
Seear et al., 2019 (63)	Australia, *(Community Health Centre)*	To understand programme acceptability and feasibility	Aboriginal people at high risk of T2DM: (n=6) Female 3, Male 3 Age: 18-24	**Derby Aboriginal Health Service Intervention**; *-Facilitated DSME sessions (exercise circuit, educational topics, outdoor cooking) over 8 weeks*	Semi-structured Interviews; Comments on teaching resources and changed health behaviours	Participants made changes in behaviours (e.g. shopping choices, portioning and soft drink consumption). Limitations were programme timing and competing demands.
Shepherd et al., 2014 (52)	USA, *(Participants’ homes)*	To describe participant experiences of a CHWP-led, household-level intervention	Adults with T2DM or pre-diabetes: (n=40). 32 Female, 8 Male Age: 26-83 Race/ethnicity: 100% Hispanic	**The Hispanic Diabetes Education and Prevention Project**; *-Delivered DSME group sessions, Encouraged family participation* *-Used incentives (pedometer, glucose monitor, tailored cookbook*	40 semi-structured Interviews; Thematic Analysis Exploring increased knowledge, social support, household-level change, and skill building	Promotoras provided valuable social support and encouraged behaviour change by building relationships based on trust and cultural understanding. Involving families was valuable.
Shiyanbol et al., 2022 (58)	USA, *(Telephone)*	To assess the acceptability and feasibility of Peers EXCEL	African Americans with T2DM: (n=8) Female 7, Male 1 Average age: 54	**Peers ECXEL**; *-6 phone support calls to share successful DSME strategies over 8 weeks* *-Phone calls followed 2 diabetes educator and pharmacist-led group sessions*	Semi-structured interviews; Content Analysis Exploring depression, emotional support and, cultural tailoring	Perceived improvements in provider communication, goal setting strategies, motivation, and confidence for self-management.
Sinclaire et al., 2020 (64)	USA, *(Community Venues)*	To evaluate the outcomes of a culturally tailored diabetes self-management intervention delivered by peer educators to Native Hawaiian and other Pacific Islanders (NHPIs)	NHPIs with T2DM: (n=32); 24 Female, Male 8; Average age: 55 Race/ethnicity: NHPIs	**Partners in Care Intervention**; *-Led DSME group sessions* *-Used incentives (exercise band, dosette box)* *-Worked alongside registered dietician*	3 Focus Groups; Thematic Analysis Exploring appropriateness, acceptability, adoption, and fidelity.	Participants valued educational materials, hands on activities, cultural aspects of the programme (e.g. stories and analogies), and group format.
Turner et al., 2021 (59)	USA, (Telephone)	To identify the key themes experiences of men who participated in the intervention	Veteran men with T2DM: (n=14) Age: NR Race/ethnicity: 10 African American, 4 white	**Technology-Enhanced Coaching program**; *-6 months* *- Helped patients establish health goals and over 6 months and assisted in creating action plan*	Semi-structured Interviews; Grounded Theory exploring shared diabetes experience with CHWP, supportive CHWP, accountability, and helpful tips	Participants engaged with intervention as CHWPs were supportive, and shared common experiences.

^a^NR, Not Reported. DSME: dietary & exercise advice, improving healthcare access; Community Venues: Library, Places of Worship, Sports centres, Community Halls.

### 3.2 Qualitative synthesis

The TFA as developed by Sekhon et al. has been refined to bring greater clarity and concision: the domain ‘*Ethicality’* has been renamed *‘Cultural sensitivity’*; a phrase more precisely describing the domain and its constructs as represented in this review. Due to overlaps in the findings, *‘Burden’* and ‘*Opportunity Costs’* were grouped to form one domain as were the related domains of *‘Intervention Effectiveness’* and *‘Self-efficacy’* resulting in five final domains and a total of 14 constructs. **Table 2** presents definitions of the main domains and emergent constructs.

**Table 2:** Mapping of domains, defined constructs and emerging findings.

Domain	Definition	Construct	Definition
1.Affective Attitude	How participants feel about CHWP-led interventions	**1.1** Satisfaction with CHWPs	Overall satisfaction with the intervention including CHWPs’ knowledge and abilities
		**1.2** Patient-CHWP relationship	Participants views on and personal experiences with CHWPs
		1.3 Attitudes to standard healthcare	Issues of trust and the degree involvement of other HCPs in delivering the intervention
2.Burden/Opportunity Costs	Perceived amount of effort required to participate/Benefits or values that must be given up to enable participation	**2.1** Health Implications	The impact of physical or mental health on ability to participate
		**2.2** Personal Commitments	External responsibilities which affect intervention participation including work and childcare
		**2.3** Logistical Barriers	The resources required for intervention participation including transport to venues, cost of food, and local facilities
3.Cultural sensitivity	How well intervention aligns with individual beliefs and values	**3.1** Cultural beliefs	Gendered roles and attitudes
		**3.2** Cultural awareness of CHWPs	Understanding of customs and traditions (and implications for diet)
		**3.3** Language	Delivery of the intervention in multiple languages
4.Intervention Coherence	How far participants understand CHWP interventions and their construction	**4.1** Content of intervention	Diabetes self-management and education (DSME) relating to the skills and strategies to manage diabetes and health consequences
		**4.2** Methods and materials	The range of teaching aids used including visual techniques and supportive materials
		**4.3** Structure	Duration, the use of group and one-to-one sessions, telephone coaching, and support of co-participants
5.Effectiveness and self-efficacy	How well the intervention worked and participant’s ability to sustain self-management	**5.1** Changed health behaviours	Impact of interventions on a range of self-management and lifestyle behaviours
		**5.2** Increased acceptance and self-confidence	The confidence and self-esteem when independently managing diabetes in developing self-efficacy and acceptance

HCP, Healthcare Professional; CHWPs, Community-based Health Workers and Peers.

Below we explore the findings within each domain and construct, using exemplar participant quotes (shown in italics). Participant characteristics, where available, are provided to contextualise their views. (Male and female Latino and Hispanic participants are referred to hereon as “Latino(s) or Latina(s)”).

#### 3.2.1 Affective Attitude

##### 3.2.1.1 Satisfaction with CHWPs

Participants described their overall appreciation for the involvement of CHWPs in terms of their communication style and the way in which they made participants feel at ease (48–53, 55, 60–65). As one African American participant described:

*“My experience was really good. I mean, everybody made me comfortable … as far as the way they explained things, especially in the meetings … it was easy to follow along.” [African American Participant]* (58)

In another example, a participant described how the CHWP recapped the basics of healthy eating without judgement after a participant was witnessed snacking unhealthily:

*“When I first started with Ms. … I was sneaking eating and she would know it. One time she caught me. She did not get all aggressive or ugly like that. She just broke it down to me and brought it down to how important my health was and got me back on the right track.” [African American participant]* (56)

Despite the generally positive response towards CHWPs, participants across several studies were dissatisfied with CHWPs’ clinical knowledge, and inability to answer more detailed questions (51, 60, 62, 64, 65). As one participant described:

*“I think … some things that maybe they couldn’t really give you the answer to, I think they should know a little more … I think we asked her something one time and I can’t remember what it was but she couldn’t really answer either.”* (60)

Related to this, CHWP training varied from a brief induction delivered by research staff (58) to an in-depth training programme with regular updates delivered by local health care providers (61). In studies the training regime was often poorly described or inconsistent making it difficult to evaluate the resulting impact on intervention quality (49, 52, 55, 56, 60, 63).

##### 3.2.1.2 Patient-CHWP relationship

Participants in four studies described the trusting relationship they developed with CHWPs and their ability to engage in open discussions about their success and failures following CHWP guidance (59–61, 66). As one African American participant explained:

*“With him [CHWP], I could keep it real. I could tell him I didn’t do this or that.” [African American, Male]* (59)

This was contrasted with feelings of being judged by clinicians. As a Latino participant in the USA described:

*“The doctor sees my blood tests and he tells me it’s bad and that I’m not doing well. That makes me feel worse. She [promo-tora] doesn’t judge us.” [Latino participant]* (50)

This apparently unconditional support contributed to some participants describing their relationship with CHWPs as akin to that of a senior family member (50, 61). As one participant in a study conducted amongst the Navajo population in the USA, described:

*“I can tell her anything about my personal life … [she] is like my grandma and [she] is like a cousin sister to me.” [Navajo, male]* (61)

The trusting, respectful nature of this relationship led to CHWPs exerting greater levels of influence. As exemplified in the comments of a Latino participant in a study in California:

*“About two weeks went by and I still didn’t feel like making changes. I kept wanting to eat the same things, but the encouragement that the [*promotora*] gave me … That was a source of motivation.” [Latino participant]* (51)

In relation to the importance of shared characteristics, participants in three studies described the significance of the CHWP having diabetes (50, 60, 65). Latino and African American participants in two studies felt that CHWPs who did not have diabetes could not fully understand their feelings as they lacked experiences of the challenges presented by T2DM (50, 65).

##### 3.2.1.3 Attitudes to standard healthcare

Participants in four studies, which included Native American, African American, and Latino populations, described their negative perceptions of health care professionals (HCPs) (54, 60, 61, 67). In one study set in Latino communities, doctors were perceived as lacking “*personalismo*”; the belief within Latino culture of being able to personally relate to others regardless of social or economic standing (67). As one participant explained:

*“There is no such thing as ‘personalismo’ anymore. Doctor’s take so little time with us…” [Latino participant]* (54)

There was also a lack of trust in the judgement of clinicians, for example one Native American participant raised concerns on the over medicalisation of diabetes:

“*What if you’re at the normal level again and doctor just keeps telling you, take it, take it?” [Navajo, male]* (61).

There were also concerns that clinical staff were formerly connected or answerable to central government organisations and authorities. One Latina participant in a study set in Detroit, USA described how she felt threatened by doctors as she believed that professional workers would send her to the police or the Immigration and Naturalization Service (60).

Despite the reservations that some held, two interventions where CHWPs worked alongside other health (and social) care providers were well-received by participants (51, 53). In the study set in the Latino population, one participant described how they were motivated to adhere to the intervention by the additional *gravitas* of a clinical opinion:

*“My physician explained to me that I needed to take the program very seriously since I had a high A1c level, that’s when I took it seriously.” [Latina participant]* (51)

#### 3.2.2 Burden/opportunity costs

##### 3.2.2.1 Health implications

The physical and mental health of participants related to T2DM severity affected ability to attend sessions in two studies set in Latino populations (49, 51). As one participant from a Latino population in Connecticut, USA explained:

*“Sometimes I’m not feeling well, sometimes I feel lightheaded or have a headache related to my diabetes, so I have to cancel my session…’’ [Latino participant]* (49)

In this instance, participants favoured programme adaptations which facilitated remote access and therefore minimised the impact of ill health on the ability to travel. As one participant explained:

*“I would prefer to meet with the CHW more often at my place, [because of] my health conditions It’s sometimes difficult for us to meet in person at the clinic.” [Latino participant]* (49)

##### 3.2.2.2 Personal Commitments

A number of participants described the difficulties in attending classes due to a range of personal obligations and responsibilities. This included a lack of childcare or childminding facilities (63) and work commitments (49, 58). In particular, the scheduling of sessions immediately after office hours impacted attendance. As one African American participant explained:

*“… I get off work at 5:00…I work on the phones, and I’m quiet for hours afterwards. I just don’t want to hear myself talk.” [African American participant]* (58)

##### 3.2.2.3 Logistical barriers

Participants described a number of logistical barriers that inhibited sustained engagement with the intervention specifically relating to transport issues, expense, and local amenities (49, 51). In Latino communities in Connecticut, USA, one participant described impact on their engagement with the intervention due to difficulties in reaching the venue of the intervention:

*“My weight is still fiuctuating, sometimes I lose weight, then I gain it back because it’s hard for me to get to the YMCA.’’ [Latina participant]* (49)

Latino participants in California, USA described their frustration at the costs associated with healthy eating and purchasing the fresh food recommended (51).

*“Sometimes the reality is that organic and healthy food are expensive.” [Latina participant]* (51)

Participants in the same intervention received advice from CHWPs that walking was a simple but effective way of increasing physical activity (51). However, the high levels of violent crime in the local neighbourhood limited opportunities for outdoor exercise:

*“One goes to the park to exercise and … things happen … well, one gets assaulted. Then it’s scary.” [Latina participant]* (51)

#### 3.2.3 Cultural sensitivity

##### 3.2.3.1 Cultural beliefs

The impact of pre-existing cultural mores on engagement with the intervention were described in Latino populations in two studies based in the USA (51, 54). Female participants reported difficulties in changing their diet due to resistance from their partners and children as refusing to cook traditional dishes was considered culturally disrespectful (51, 54). This could lead to family conflict and drove some Latina participants to abandon the programme (51). The cultural concept of *‘machismo’* holds that men are tough and possess a superior position as head and breadwinner of the family (68). This led to marital conflict where some husbands objected to their wives attending classes:

*“This was about to cost me a divorce. Because I would go to classes and my husband didn’t like it.” [Latina participant]* (51)

It also meant that men faced pressure to maintain a strong image. For example, in a study based in Texas, USA, men described their reluctance to share personal challenges with CHWPs in group sessions:

*“I keep my mouth shut; it is not macho [manly] to tell someone that you are weak. Mexican-American men just don’t feel comfortable sharing their weakness in public.” [Latino participant]* (54)

##### 3.2.3.2 Cultural awareness of CHWPs

CHWPs shared or understood the cultural characteristics of participants which helped contextualise their advice and guidance (50–54, 60–62, 64). For example, they were able to describe how familiar foods could be used in controlling blood sugar (54, 62). As one Latina participant from a study set in Texas, USA described:

*“Just knowing how I can eat a little bit of menudo [traditional Mexican soup], makes me feel less restricted. I always test afterwards now to see how me and the menudo did.” [Latina participant]* (54)

Participants also appreciated CHWPs’ awareness of hospitality customs which present additional dietary temptations and social pressure, from well-meaning hosts (54, 62). CHWPs gave individuals the confidence to politely decline some of the foods offered, as one Punjabi participant explained:

*“I learned to say no at social gatherings i.e., no samosas!” [Punjabi participant]* (62)

##### 3.2.3.3 Language

It is widely understood that bilingual services can further increase the cultural applicability of self-management interventions (69) and the ability of CHWPs to speak the same language as participants was not only appreciated by participants but in was some instances a necessity (50, 62). As one Latino participant in Pennsylvania, USA described:

*“Some of us don’t speak English, so it is important to have the classes in Spanish.” [Latino participant]* (50)

Educational materials should also be translated into the native language of participants as partial translation can create a barrier for monolingual participants (62). One Punjabi participant noted this barrier when some intervention components were only available in English:

“Please put all information about Leader’s Manual on website and next workshop dates information in Punjabi.” *[Punjabi participant]* (62)

#### 3.2.4 Intervention coherence/effectiveness

##### 3.2.4.1 Content of intervention

Participants described how they had an increased understanding of the link between activity, diet and blood glucose control due to practical and applied examples used by CHWPs (48, 50–53, 57–63, 65). As one Hispanic participant from a study set in Washington, USA explained:

*“I was always scared about how, if I eat this food, ah, my sugar would go way up and stuff. But [the promotora] taught me that you can eat that food, just not a lot of it.” [Hispanic participant]* (52)

The content of the interventions included advice on physical activity, cooking guidance, and elements of clinical management (49, 52, 57, 58, 61–64).

##### 3.2.4.2 Methods and materials

A range of teaching materials were employed including Loteria (teaching cards) (51); self-completed workbooks (54); visual props (61, 63) including photovoice, a process that uses participant-taken photos to encourage reflection on community needs and concerns (55). As one Native American participant described:

“*She brought pictures and I understood from those pictures, including the flipcharts.” [Navajo, Female]* (61)

Two different interventions incorporated practical cooking sessions (57, 63), and one of these also included an exercise circuit (63). The practical application of this information was helped by the distribution of supportive materials for healthy eating such as cookbooks (52), measuring cups, and portioning plates (61). To encourage physical exercise, pedometers (52) and exercise bands (61, 64) were supplied, and to support clinical management of T2DM dosette boxes (64) and glucose monitors (52) were provided.

##### 3.2.4.3 Structure

CHWP-led interventions utilised group sessions (48, 50–52, 60, 62, 64); one-to-one sessions (61, 70); or a combination of both (57, 58). Participants in four studies favoured group sessions as it allowed them to form friendships and expand their social network, particularly where support was unavailable at home (49, 50, 65, 66). As one Latina participant from Connecticut, USA explained:

*“I live alone so sometimes I feel isolated. However, now that I’m in this program, I feel more connected.” [Latina participant]* (49)

Participants also valued hearing others speak about their personal challenges with diabetes which served to reassure them that others faced similar obstacles. As one participant explained:

*“You feel a little more comfortable with somebody that’s going through the same thing that you are going through.” [African American participant]* (58)

Another participant described how their CHWP had actively facilitated and encouraged participants to connect and support one another as they undertook the intervention:

*“The promotora kept us motivated by encouraging us to exchange phone numbers, telling us to call other classmates when we didn’t feel well, explaining how we felt; and that helped us to open up on many things.” [Latino participant]* (51)

The structure of the various interventions frequently lacked detail on duration but in two studies participants expressed regret that they did not last longer (48, 49). Elsewhere, participants wanted more emphasis on follow-up between classes to consolidate learning and offer additional support (57, 62, 64). There was also a desire to retain access to CHWPs for advice following completion of the intervention. For example, one African American participant explained how this contact would help negotiate the Christmas holidays:

*“December’s coming up … I do remember we talked about, you know, dealing with depression, holidays, meals, and things like that. I would like to get a brush up on that…” [African American participant]* (58)

The majority of the interventions were conducted entirely face-to-face though three studies incorporated follow-up telephone calls in between taught sessions (53, 56, 65). As one participant explained:

*“*They always concerned about different things like your sugar, your HbA1C, are you keeping up with those type of issues and that helps because I never been in that space where people call me to see how I’m doing and am I doing the correct things and its pleasurable to get that kind of attention.” *[African American participant]* (56)

#### 3.2.5 Effectiveness and self-efficacy

##### 3.2.5.1 Changed health behaviours.

Participants in a number of studies reported improvements in a range of self-management behaviours as a result of the CHWP-led intervention (48, 50–53, 57, 59–63, 65): As one Latino participant described:

*“I have a better understanding of how to test my blood sugar, how to manage my diabetes, navigate my different medical appointments, and also engage in more physical activity.” [Latino participant]* (49)

Participants described how they effectively applied CHWP teaching to transform their diet (52, 61). As one Native American participant explained:

*“I totally got away from sodas. I don’t drink sodas no more. Bread, I don’t eat no more. Just water, constantly, every day, I drink a lot of water. Junk food I don’t eat anymore.” … “Even when we eat out, my family, I just get a salad while they eat their burgers.” [Navajo, male]* (61)

A Latino participant in a study set in Washington, USA described how the CHWP intervention helped change the eating behaviours of their entire family (52):

*“We didn’t have a good habit and … they would eat at a certain time and the kids would be snacking at a certain time and, ah, well, now it’s like “No! We’re gonna get breakfast, lunch, and dinner and we’re gonna sit down - we’re gonna do this together!’ “ [Latina participant]* (52)

Two different studies explored the introduction of exercise and physical activity into daily routines with participants reporting positive effects the interventions had on their routine levels of activity (61, 62). In a study set amongst the Punjabi community in Canada one participant described how they made a conscious choice to use the stairs instead of the elevator:

*“I used to walk only with a walker but now am improving and am walking with a cane, I climb the stairs now for exercise and I’m getting stronger.” [Punjabi participant]* (62)

Also related to increased activity, a Native American participant described how following the intervention they became more active, whether in the home or through outdoor exercises such as taking a walk:

*“Instead of staying on the couch, do something around the house, she said. At least take a walk to the highway and come back or walk around the house.” [Navajo, female]* (61)

Another participant in the same study described how they more regularly monitored blood glucose as encouraged by the CHWP which confirmed that their efforts were succeeding:

*“I’ve been improving … I check my blood all the time and, uh, pretty good. Down to 98, 96.” [Navajo, male]* (61)

##### 3.2.5.2 Increased acceptance and self-confidence

Following programme completion, participants in a number of studies described how they felt empowered and better prepared when communicating with HCPs (48, 52, 53, 60, 62, 65). As one participant from Detroit, USA stated:

*“Now I feel more comfortable talking with my doctor and asking all that I think that I need to know. I know more, so I can ask more. I’m not so afraid.” [African American participant]* (60)

Another African American participant expressed similar feelings of increased confidence when interacting with clinicians and appreciated that they have a responsibility to help patients:

*“…you do not have to be afraid. Just you are [in] there, that’s your doctor. It’s confidential. Whatever it is, whatever you feel, that’s what they are there for.” [African American participant]* (65)

Participants in a number of studies described how the intervention helped them come to terms with their diagnosis and were therefore psychologically better equipped to live with diabetes (50, 51, 54, 60, 62). As one Latino participant explained:

*“My lifestyle has changed a lot. I feel happy and relieved. Before, I felt bad. But now I have more self-esteem. Before, I thought that there was no solution. Now I know that I can live well and feel well.” [Latino participant]* (50)

## 4 Discussion

### 4.1 General findings

Our research provides a much-needed synthesis of primary qualitative studies exploring CHWP-led interventions designed to improve self-management of T2DM within ethnic minority groups. The TFA has proven a useful tool in exploring existing evidence with pertinent findings emerging within each of the five domains. *Affective attitude* described participants’ overall positive attitudes towards CHWP-led interventions. Participants responded favourably to CHWPs as their shared characteristics enabled a closer and more familial relationship in comparison to mainstream health care providers, though not without concerns over the depth of their clinical knowledge. In considering *Burden* and *Opportunity Costs*, participants reflected on the difficulties faced attending and completing the interventions. These included the impact of poor health, cost, and obligations of work and family. In relation to *Cultural sensitivity*, participants appreciated the enhanced understanding of their needs and challenges demonstrated by CHWPs including how they placed strategies for behavioural change in a culturally-relevant context. The evidence related to *Intervention Coherence* indicated that participants responded positively to the content of DMSE, the camaraderie and support with fellow participants developed during group sessions, and interventions involving telephone follow-up. Finally, in examining the impact on *Effectiveness and Self-efficacy*, participants described sustained changes to health behaviours and the increased confidence in managing their condition and interacting with senior clinicians. Below we discuss these findings in more detail and conclude with a series of recommendations based on the presented evidence to inform future CHWP-led interventions (28).

### 4.2 Specific findings

#### 4.2.1 Affective attitude

Participants responded positively to the delivery of self-management interventions by CHWPs (50–52, 54, 58, 60, 61), though in some studies participants highlighted concerns over the limitations of CHWPs’ clinical knowledge (60, 62). The structure of CHWP training varied in the studies identified but concerns over the level of clinical knowledge of CHWPs have emerged previously in a range of interventions. The lack of focus on CHWP training has been identified previously in other conditions (71) as well as in T2DM interventions (72, 73), resulting in a call for additional assistance or training (74).

It is understood that a trusting relationship between patient and HCPs encourages personalised care and improves overall patient experience (75–77). Establishing such trust is especially important in improving engagement for marginalised communities often wary of traditional healthcare organisations due to previous negative experiences such as language barriers, poor cultural competence and racial discrimination (78–81). Contrary to this suspicion of HCPs, the studies we identified described the close bond created between participants and CHWPs, crediting their shared socio-cultural background (49–52, 58–61). There is also increasing evidence that through their role as mediators and advocates, and an increased emotional understanding, CHWPs can help restore patient trust in the healthcare system (82–87).

#### 4.2.2 Burden/opportunity costs

A range of factors relating to personal circumstances including poor health, work, logistical barriers relating to travel, and the cost of fresh food impacted engagement with the intervention (48, 49, 51, 56, 63–65).

The issues identified around transport and work commitments are also widely recognised in the general population with T2DM (88–90). Although these problems are not specific to minority populations (91), in USA and Europe they are more prevalent in these groups due to the intertwined relationship between ethnicity and socio-economic status (92). Lower socio-economic status amongst minoritized populations is reflected in longer commutes, use of public transport and multiple low-paid employment, all of which impact ability to attend self-management interventions (92–94). The prohibitive cost of “healthy” eating also reflects existing evidence of the negative impact of food insecurity on controlling diabetes in minority populations (95–97). Solving these broader socio-economic determinants is difficult within the scope of a single intervention but there are indications that providing transport (98), food coupons (99), or “food is medicine” type initiatives where nutritional food is prescribed by HCPs (100) can help low-income families manage diabetes (101). There is also the potential of CHWPs to visit individual households or provide remote sessions as ways of encouraging engagement with self-management support (102) though these are subject to issues of connectivity and digital literacy which can be limited in minoritized populations (103).

#### 4.2.3 Cultural sensitivity

There are a range of culturally specific influences that can impact healthy behaviours in minoritized populations including gendered roles, religion, and custom (104). Implementing dietary changes for those with T2DM is challenged both in the USA and Europe by strong hospitality cultures, particularly during traditional religious celebrations or personal events (105–109). In this context, the culturally sympathetic dietary advice delivered by CHWPs familiar with traditional foods and social pressures was highly valued by participants (50, 52–54, 60, 62, 64).

It is also broadly understood that trends in health behaviours are closely tied with established cultural beliefs around family and gender (104). This appeared particularly pronounced amongst Latino participants in the studies we identified (51, 54). In Latino populations this is exacerbated by strongly gendered roles for men and women (68, 110–114). In Latino communities, delivering dietary interventions at a family-level has been identified as one strategy to overcome domestic resistance (115) and have proven successful in preventing T2DM (116), and promoting a range of improved self-management behaviours in Latino adults diagnosed with T2DM (117, 118).

The ability of CHWPs to deliver interventions in the native language of participants was key for some Latino, Navajo and Punjabi participants (50, 61, 62). This has been previously recognised as an important consideration in delivering self-management interventions in Latino (119) Black African and Caribbean (120), Asian (121) and a range of minority populations (122, 123). However, providing content in a familiar language does not itself overcome issues of health literacy which is typically more predominant in minoritized populations (124). Tailoring health information to individual levels of understanding is required in both minority and general populations (125) and there is evidence that comprehension can be improved by their co-production (126–128).

#### 4.2.4 Intervention coherence

The applied content of the DSME delivered by CHWPs in the identified studies were credited by participants with effectively linking theory to practical self-management advice (48, 50–53, 57–63, 65). This included how to interpret food labels, and the relationship between blood sugar levels and nutrient intake (57, 63, 64) (49, 52, 58, 61, 62). This reflects previous evidence that the content of CHWP-led T2DM interventions in the general population, particularly where they combine multiple teaching strategies, are considered more practical than traditional hospital consultations (129–137).

A mixture of teaching materials were used to mitigate differences in health literacy including: visual aids such as photovoice (55) previously recognised as an effective tool in conveying needs of individuals and their communities (138, 139), ‘*loteria cards’* (51), and workbooks (54) which have also proved successful in DSME for Black women with T2DM (140). There is also evidence from previous DSME programmes that including practical sessions such as interactive cooking classes can help those with diabetes better manage HbA1c (141–143).

Non-judgemental support provided by either class peers or CHWPs, in particular where CHWPs also had diabetes, was well received (48, 49, 51, 58, 59, 62). CHWPs’ follow-up telephone calls between sessions were welcomed and regular provider contact has previously been associated with increased engagement with self-management regimes in general populations (144).

First-hand accounts of the challenges and consequences of (uncontrolled) T2DM whether from peers or CHWPs resonated with participants (51). This echoes previous work that described the motivation provided by improved awareness of serious diabetes related complications within the general population (145). The importance of the peer support from fellow participants was also highlighted and again these benefits have been observed in minoritized populations with T2DM (15, 146, 147) as well as in the general population (148).

#### 4.2.5 Effectiveness and self-efficacy

Participants described improvement in a number of self-management behaviours including increased physical activity and healthier dietary habits, demonstrated in several studies by improved HbA1c control (48, 50–53, 57, 59–63, 65). CHWP-led interventions have proven similarly effective in the general population (73) with growing evidence of significantly improved blood glucose control (149, 150).

Individuals from all populations require a high level of self-efficacy to successfully manage the complex demands of T2DM self-management (151, 152). Participants in a number of identified studies reported greater confidence regarding independently managing diabetes and notably in their interactions with HCPs, where they were encouraged to be more active in discussions with clinicians. For example, questioning whether they had access to the necessary diabetes services and tests and querying any treatment changes (50, 58, 60, 62). Effective coaching on communication with HCPs is an increasingly important element of DSME programmes aimed at the whole population (153) but especially in minority groups where there are long-standing issues of patient passivity and miscommunication (154).

### 4.3 Strengths and limitations

This comprehensive review provides valuable insight on minority perspectives of CHWP-led interventions and shared perspectives within the general population (10, 12–14). They also highlight that utilising CHWPs might mitigate some of the issues around language and culture but these interventions are still vulnerable to broader socio-economic issues that can impact attendance, and sustained engagement (13, 155). The TFA proved a useful means of organising the data and its comprehensive structure added to the reviews methodological rigour which used best practice throughout (34, 47, 156). However, the overall quality of the studies was low, their design heterogenous and the specifics of the intervention were often poorly described (49, 52, 55, 56, 60, 63). One element shared by all studies was that they assessed participant experience retrospectively introducing the possibility of recall bias (157). As with other reviews, it is also vulnerable to the biases introduced by the authors of the original studies (158) and we discovered a lack of overall study quality which has been reported in similar reviews of CHWP-led interventions (159).

Although the majority of studies are USA-based, and conducted in Latino and African American populations the work also includes valuable yet underrepresented perspectives of Native Americans, Punjabis and Aboriginal people (160, 161). However, caution should be applied when considering these findings in relation to other ethnicities and minoritized populations should not be considered a homogenous group and individualised co-designed approaches would ideally be pursued.

The majority of the CHWPs studied were female and though this is reflective of the CHWP workforce (with recent estimates suggesting 70% are female) (162), there may be gender specific responses related to male CHWPs that require further enquiry. A large number of studies were excluded because the CHWP element was part of a larger, multi-component self-management support programme. Future work might more closely observe the impact of the CHWP-element of any broader programme including exploration of the CHWP’s perspective.

### 4.4 Implications for future practice

Despite the clear potential of CHWP-led interventions to address some of the persistent challenges faced in improving T2DM self-management both in general and minoritized communities, there is still the opportunity for improvement in design and delivery. This includes exploring the experiences of lower- and middle-income countries in their utilisation of CHWPs. Below we draw out some practicable recommendations that might be applied when introducing CHWP-led interventions, presenting each within the domains of the TFA. These considerations alongside the broader findings of our review have been used to inform a series of questions for those commissioning and implementing CHWP-led interventions in ethnic minorities (see **Supplementary File 3**). Future programmes and research might draw on these, to improve practice and expand the non-professional health workforce.

#### 4.4.1 Affective attitude

Training and coordinating CHWPs for these roles is crucial and requires further research (73). There is opportunity to link CHWPs more closely with multidisciplinary teams who can provide ready access to more specialised clinical expertise (163). There is existing evidence that suggests a need for more systematic and comprehensive CHWP training, whether delivering interventions to minorities (72) or the general population (73, 164, 165).

It has been acknowledged that CHWPs can act as cultural mediators, liaison workers, navigators or advocates and their role covers various aspects of social support and health education (38). In identified studies CHWPs were employed in a number of varying roles however it is key that the precise roles of CHWP are understood and the expectations of participants and commissioners are established.

#### 4.4.2 Burden opportunity costs

Many of the barriers associated with attendance and adherence to CHWP-led interventions in minorities are more broadly applicable across the general population (10, 12–14). To accommodate working adults and those with limited transport access or poor health, interventions should be available in a range of remote formats including online platforms and telephone coaching (166). There should also be a general focus on more readily accessible community venues with flexibility around timing (150, 167, 168). Issues of food security should be acknowledged by CHWPs in the content of the intervention and to help address this participants should be linked with support mechanisms through the local government or healthcare services (99–101).

#### 4.4.3 Cultural sensitivity

Family-level interventions can potentially mitigate the influence of culturally-gendered roles and should therefore be considered in male-dominated cultures (117, 118). The CHWP workforce is also predominantly female hence it is vital to consider the recruitment of male CHWPs to support male participants that may otherwise feel inhibited by female leads (162, 169).

#### 4.4.4 Intervention coherence

To help combat barriers of language and (health) literacy, CHWPs should ideally speak the same native language as participants and use educational materials written plainly and preferably co-designed with target populations (15, 170–174). In considering content, participants should be provided with practical information on how to marry cultural behaviours or practices with a healthy lifestyle (52, 61, 62, 175, 176).

#### 4.4.5 Effectiveness and self- efficacy

The benefits of interacting with other culturally similar participants were described and more formally facilitated links between participants might be considered in future CHWP-led interventions (146, 177). Also important was coaching participants in how to effectively communicate with HCPs. This strategy has been successfully incorporated into a number of self-management interventions for T2DM and would ideally also be more universally applied to CHWP-led interventions (178).

## 5 Conclusion

This review has highlighted the key factors influencing how minoritized participants perceive CHWP-led interventions. Programmes were mostly positively received but points contributing to high attrition rates were raised. Many of the described barriers are contextual and also impact the general population undertaking T2DM self-management support interventions signalling a need for broader consideration at a health economics and policy level. However, barriers were often heightened in minoritized populations indicating that there are areas where the content or delivery of the intervention can be improved or more finely attuned to the socio-economic contexts of specific minoritized populations. Related to these we have specified a number of elements that warrant consideration for those commissioning or delivering these interventions.

## Data availability statement

The original contributions presented in the study are included in the article/**Supplementary Material**. Further inquiries can be directed to the corresponding author.

## Author contributions

VG: Conceptualization, Formal analysis, Project administration, Writing – original draft, Writing – review & editing, Data curation. IL: Conceptualization, Formal analysis, Project administration, Writing – original draft, Writing – review & editing, Methodology, Supervision.
