# Supplementary material for: Acceptability of community health worker and peer supported interventions for ethnic minorities with type 2 diabetes: a qualitative systematic review
Source: Front Clin Diabetes Healthc. 2024 May 21;5:1306199. doi: 10.3389/fcdhc.2024.1306199 (PMC11148349; doi:10.3389/fcdhc.2024.1306199)
Supplement: Supplementary file 2 [file Table_2.docx]

**Supplementary File 1**: Eligibility Criteria using the SPIDER (Sample, Phenomenon of Interest, Data, Evaluation, Research Type) tool

| **SPIDER** | **Inclusion** | **Exclusion** |
| --- | --- | --- |
| Sample | - Adults (≥18) - Have T2D - Ethnic minority | - <18 - Studies focussing on patients with gestational diabetes, T1D or any other cause - Programmes where ethnic minority adults make up <50% of sample |
| Phenomenon of Interest | - CHWP-led/Co-led programme - Conducted in Canada, New Zealand, Australia, Europe or the US | - Complex interventions, where the role of the CHWP cannot be clearly identified |
| Design | - Any qualitative method (e.g. focus groups, interviews, open-ended surveys) - Published peer-reviewed studies or grey literature (e.g. dissertations and theses, research reports) | - Quantitative methods |
| Evaluation | - Considering the experiences and perspectives of patients | - Only considers experiences and perspective of CHWPs - Only includes quantitative outcomes |
| Research type | - Qualitative or mixed method | - Only includes quantitative analysis - Cannot isolate qualitative analysis in mixed methods - Reviews (e.g. literature, scoping, systematic) |
