# Supplementary material for: Acceptability of community health worker and peer supported interventions for ethnic minorities with type 2 diabetes: a qualitative systematic review
Source: Front Clin Diabetes Healthc. 2024 May 21;5:1306199. doi: 10.3389/fcdhc.2024.1306199 (PMC11148349; doi:10.3389/fcdhc.2024.1306199)
Supplement: Supplementary file 3 [file Table_3.docx]

**Supplementary File 2:** Example Medline Search

1. ((("semi-structured" or semistructured or unstructured or informal or "in-depth" or indepth or "face-to-face" or structured or guide) adj3 (interview* or discussion* or questionnaire*)) or (focus group* or qualitative or mixed method*)) mp. or focus groups/ or narration/ or qualitative research/ or view*.mp. or experienc*.mp. or opinion*.mp. or attitude*.mp. or perce*.mp. or perspective*.mp. or belie*.mp. or feel*.mp. or know*.mp. or understand*.mp.
2. Community Health Workers/
3. ((lay adj worker?) or (lay adj health* worker?) or (lay adj health care worker?)).ti,ab.
4. (lay or peer adj3 (counselor? or counsellor? or counseling or counselling or coach* or intervention? or support or outreach or delivered or staff or led or provider? or based or volunteer? or mentor* or educator? or navigator? or adviser? or advisor? or facilitator? or leader* or person*)).mp.
5. (community worker? or community health* worker? or community health care worker? or community volunteer? or Promotora de Salud or promotora).ti,ab.
6. (community based worker? or community based health* worker? or community based health care worker? or community based volunteer?)mp.
7. ((nonprofessional? or nonprofessional? or paraprofessional?) adj3 (counselor? or counsellor? or counseling or counselling or coach* or intervention? or support or outreach or delivered or staff or led or provider? or based or volunteer? or mentor* or educator? or visitor? or adviser? or advisor? or facilitator? or personnel)).mp.
8. (volunteer? adj3 (counselor? or counsellor? or counseling or counselling or coach* or intervention? or support or outreach or delivered or staff or led or provider? or based or mentor* or educator? or visitor? or adviser? or advisor?)).mp.
9. Or/2-8
10. Diabetes and (type 2 or type ii).mp.
11. Diabetes Mellitus, Type 2/
12. (impaired glucose tolerance OR IGT OR prediabetes or pre?diabetes).mp.
13. or/8-10
14. (ethnic minorit* or minority ethnic or ethnicity or african* or caribbean* or bame or bme or asian* or pakistani or bangladeshi or hispanic or latino or aborigin* or pacific island* or maori or american indian* ).mp. or Ethnic and Racial minorities/
15. 1 AND 9 AND 13 AND 14
