# Supplementary material for: Acceptability of community health worker and peer supported interventions for ethnic minorities with type 2 diabetes: a qualitative systematic review
Source: Front Clin Diabetes Healthc. 2024 May 21;5:1306199. doi: 10.3389/fcdhc.2024.1306199 (PMC11148349; doi:10.3389/fcdhc.2024.1306199)
Supplement: Supplementary file 4 [file Table_4.docx]

| **Authors (Date)** | **Aim** | **Method** | **Design** | **Recruitment** | **Data Collection** | **Bias** | **Ethics** | **Analysis** | **Findings** | **Value** |
| --- | --- | --- | --- | --- | --- | --- | --- | --- | --- | --- |
| Shepherd-Banigan et al. (2014)^(27)^ | Yes | Yes | Yes | Can’t tell | Yes | Yes | Can’t tell | Yes | Yes | Yes |
| Joachim-Célestin et al. (2020)^(53)^ | Yes | Yes | Yes | Yes | Yes | Yes | Yes | Yes | Yes | Yes |
| Chang W et al. (2021)^(25)^ | Yes | Yes | Can’t tell | Can’t tell | Yes | Yes | Yes | Yes | Yes | Yes |
| Deitrick LM et al. (2010)^(26)^ | Yes | Yes | Yes | Yes | Yes | Yes | Yes | Yes | Yes | Yes |
| Sinclair KA et al. (2020)^(54)^ | Yes | Yes | Can’t tell | Yes | Yes | Yes | Yes | Yes | Yes | Yes |
| Heisler M et al. (2009)^(55)^ | Yes | Yes | Can’t tell | Can’t tell | Yes | Yes | Yes | Yes | Yes | Yes |
| Haltiwanger EP et al. (2012)^(56)^ | Yes | Yes | Can’t tell | Yes | Yes | No | Yes | Yes | Yes | Yes |
| Otero-sabogal R et al. (2010)^(57)^ | Yes | Yes | Can’t tell | Yes | Yes | No | No | Yes | Yes | Yes |
| Lalla A et al. (2020)^(58)^ | Yes | Yes | Yes | Yes | Yes | Yes | Yes | Yes | Yes | Yes |
| Castillo A et al. (2010)^(59)^ | Yes | Yes | Yes | Yes | Yes | Can’t tell | Yes | Yes | Yes | Yes |
| Okoro F et al. (2018)^(60)^ | Yes | Yes | Yes | Yes | Yes | Yes | Yes | Yes | Yes | Yes |
| Shiyanbola OO et al. (2022)^(61)^ | Yes | Yes | Can’t tell | Yes | Yes | No | No | Yes | Yes | Yes |
| Turner CD et al. (2021)^(62)^ | Yes | Yes | Can’t tell | Yes | Yes | No | No | Yes | Yes | Yes |
| Pullen-smith B (2014)^(63)^ | Yes | Yes | Can’t tell | Can’t tell | Yes | Can’t tell | Yes | Yes | Yes | Yes |
| Seear et al. (2020)^(64)^ | Yes | Yes | No | Yes | Yes | Can’t tell | Yes | No | Yes | Yes |
| McGowan (2013)^(65)^ | Yes | Yes | No | Yes | Yes | No | No | No | Yes | Yes |
| Jia et al. (2019)^(66)^ | Yes | Yes | Yes | Yes | Yes | Can’t tell | Yes | Yes | Yes | Yes |

***Summary File 3:*** *Summary of Quality Appraisals Using Critical Skills Appraisal Programme (CASP) Checklist for Qualitative Studies*
