# Supplementary material for: Acceptability of community health worker and peer supported interventions for ethnic minorities with type 2 diabetes: a qualitative systematic review
Source: Front Clin Diabetes Healthc. 2024 May 21;5:1306199. doi: 10.3389/fcdhc.2024.1306199 (PMC11148349; doi:10.3389/fcdhc.2024.1306199)
Supplement: Supplementary file 5 [file Table_5.docx]

**Supplementary File 4 - Questions to consider when developing CHWP-led interventions in minoritized populations**

|  | **Questions** |
| --- | --- |
| **Affective attitude**  **(towards CHWPs)** | - What does the training and ongoing support look like? - How closely are CHWPs linked with HCPs and the wider health service? - How closely do CHWPs resemble or understand the characteristics of participants?   - Religion, Language, Same health condition - Are the roles of the CHWP clearly identified? What do they include?   - Social support, health education, advocate, care navigator etc - How homogenous are the group, |
| **Burden/ Opportunity Costs** | - What is in place to support attendance?   - Does the timing of sessions accommodate shift work, long hours?   - Are childcare facilities available on site?   - What distance do people have to travel – can they be supported by the programme?   - Are there are remote elements (what is in place to support digital literacy, connectivity)?   - Are the sessions delivered in community located premises? - Are the recommendations for lifestyle changes feasible and affordable?   - What is in place to support food security/costs?   - Are there local facilities/amenities present capable of supporting increased physical activity? |
| **Cultural Sensitivity** | - What are the challenges of the gendered roles in minoritized populations? - What are the expected roles and characteristics of males and females in the family and broader community?   - Can family-level interventions be used? - Are various languages and levels of literacy accounted for? - How homogenous are participants?   - Does the intervention/identity of the CHWP account for a range of ages, gender, ethnicities or is its intention to focus on a single population? |
| **Intervention coherence** | - How is theory linked with practical strategies for self-management? - How do methods and materials accommodate a range of health literacies?   - Use of props, use of visual materials, use of practical sessions - Is specialised equipment needed to support self-management?   - Can these be provided for by the intervention, at what cost? - If using group sessions how are homogenous are the groups?   - Has attention been paid to mixture of ages, gender, and ethnicity?   - Is there the opportunity for one-2 one contact? - What support mechanisms are in place between sessions? - To what degree is peer to peer contact facilitated,   - What needs to be done to facilitate this?   - What might it look like is it online? Via telephone?   - Is it moderated? - To what degree has the content been co-produced and “owned” by target populations? |
| **Effectiveness and self-efficacy** | - What is in place to support resilience and acceptance? - What is in place to support ongoing communication with HCPs post-intervention?   - Are there strategies in place and specific coaching? |
